# Vein of Galen Malformation Manifesting as High-Output Heart Failure

**DOI:** 10.7759/cureus.20067

**Published:** 2021-12-01

**Authors:** Destiny Uwaezuoke, Andrew Wahba

**Affiliations:** 1 Pediatrics, University of Texas Health Science Center at Houston, Houston, USA

**Keywords:** endovascular embolization, cerebral angiography, vomiting, high-output heart failure, vein of galen

## Abstract

The sudden onset of vomiting in a previously healthy term neonate has a broad differential requiring a thorough history and physical examination. When this does not reveal the underlying cause, a workup must be performed to rule out potentially devastating diagnoses that must be addressed in a timely fashion. In infants, this clinical presentation could be due to infections such as sepsis or meningitis, gastrointestinal causes such as anatomical abnormalities or ingestions, or cardiac causes such as congenital heart disease. Conversely, inborn errors of metabolism or neurologic issues such as vascular anomalies or a tumor with associated increased intracranial pressure could also be the culprit. In this report, we discuss the case of a previously healthy newborn with a rare cause of vomiting and feeding intolerance, which was ultimately discovered to be due to the vein of Galen malformation.

## Introduction

The vein of Galen is a cerebral vein derived from the median prosencephalic vein of Markowski and its function is to return deoxygenated blood from the brain to the heart [1]. Anatomically, the venous drainage system is formed when the anterior and posterior choroidal arteries and the anterior cerebral artery come together to drain into a venous pouch known as the median vein of the prosencephalon. From there, blood travels into the superior sagittal sinus and then traverses a number of smaller venous systems before draining into the jugular vein and then into the right atrium of the heart [2].

Infrequently, during weeks 6-11 of fetal development in utero, an abnormality can occur within the median prosencephalic vein, and an arteriovenous shunt instead arises, which is called the vein of Galen malformation [3]. The shunt allows for more blood to flow into this vein causing its dilation. The larger the shunt becomes, the higher the preload on the right side of the heart, and, thus, these infants can enter cardiac failure. This is a rare event comprising 30% of pediatric vascular and 1% of all pediatric congenital anomalies [4]; however, it is associated with a high mortality rate if not discovered and treated in time. We present the case of a neonate with the acute onset of vomiting who was eventually discovered to have the vein of Galen malformation resulting in high-output heart failure.

## Case presentation

A 5-day-old term Hispanic male presented to the emergency room for acute onset of non-bloody, non-bilious, non-projectile vomiting after every feed. He had been exclusively breastfed with no issues for the first three days of his life. On the day prior to admission, he became tachypneic and easily fatigued while feeding. He had no reported cyanosis or fever and continued to stool normally, but only produced one wet diaper in the prior 24 hours.

He was born at 39 weeks of gestational age via spontaneous vaginal delivery to a 35-year-old gravida 3 para 3. Pregnancy was complicated by group B Streptococcus infection that was adequately treated; otherwise, it was uncomplicated newborn course with no neonatal intensive care unit stay. He was discharged on day of life 2 after passing both his hearing screen and the critical congenital heart disease screen. Of note, a fetal ultrasound was performed around 20 weeks’ gestation and there were no concerns on imaging at that time. His birth measurements were as follows: weight was 3,232 g (42nd percentile); length was 48.26 cm (41st percentile); and head circumference was 36.8 cm (81st percentile).

In the emergency room, initial physical examination revealed an oral temperature of 98.2°F, heart rate of 147 beats per minute, blood pressure of 63/33 mmHg, respiratory rate of 40 breaths per minute, and oxygen saturation of 99% on room air. Weight was 3.11 kg, with a body mass index of 13 kg/m^2^ (52nd percentile), about a 4% decrease from his birth weight. His anterior fontanel was open, soft, and flat, and his abdominal exam revealed hepatomegaly but was otherwise soft, non-distended, and non-tender. Heart examination was positive for a flow murmur and lung examination was not concerning. There was no visible jaundice, skin rash, or lesions. On a neurological examination, he was awake but irritable and was able to move all extremities equally with an intact Moro reflex. No oral ulcers, lymphadenopathy, petechiae, ecchymosis, or other signs of bleeding were present.

Initial labs revealed that he was initially normoglycemic on a point of care check, but a venous sample about one hour later showed hypoglycemia (blood glucose of 34 mg/dL). Labs also revealed hyperkalemia, hyperchloremia, hyperbilirubinemia, and transaminitis. Imaging performed in the emergency room included an abdominal X-ray and an abdominal ultrasound. Both were unremarkable, ruling out gastrointestinal obstruction and pyloric stenosis.

He was then admitted and started on phototherapy for the hyperbilirubinemia; this was discontinued 24 hours later. Subsequent labs showed persistence of electrolyte derangements and an acute kidney injury. A complete blood count showed a normal white blood cell count but 11% bandemia. His first newborn state screen resulted as positive for a potential thyroid disorder and therefore thyroid function tests were drawn and were within normal limits. On day 2 of admission, he became hypothermic to 94.8°F and developed increased work of breathing; therefore, a sepsis rule out was initiated and he was placed on supplemental oxygen via nasal cannula. Blood cultures and a urinalysis with urine culture were obtained, and a lumbar puncture was performed. He was started on empiric therapy with ampicillin, cefepime, and acyclovir. A respiratory viral panel was also obtained and was negative.

A chest X-ray showed a mildly enlarged cardiothymic silhouette with increased pulmonary vascularity prompting the need for echocardiogram. The patient required increasing levels of oxygen and was subsequently transferred to the pediatric intensive care unit due to concern of shock, possibly cardiogenic in nature. Initial lab work there showed lactic acidosis, persistent acute kidney injury, thrombocytopenia, anemia, and a B-type natriuretic peptide (pro-BNP) greater than 70,000 pg/mL. Echocardiogram revealed an enlarged right and left atrium, enlarged right ventricle, severe mitral regurgitation, moderate tricuspid regurgitation, depressed right ventricle systolic function, and elevated pulmonary artery pressures concerning for high-output heart failure and pulmonary hypertension. Due to worsening respiratory failure, he required intubation, and a head ultrasound was performed to rule out a neurologic issue as a cause of his presentation.

**Figure 1:**
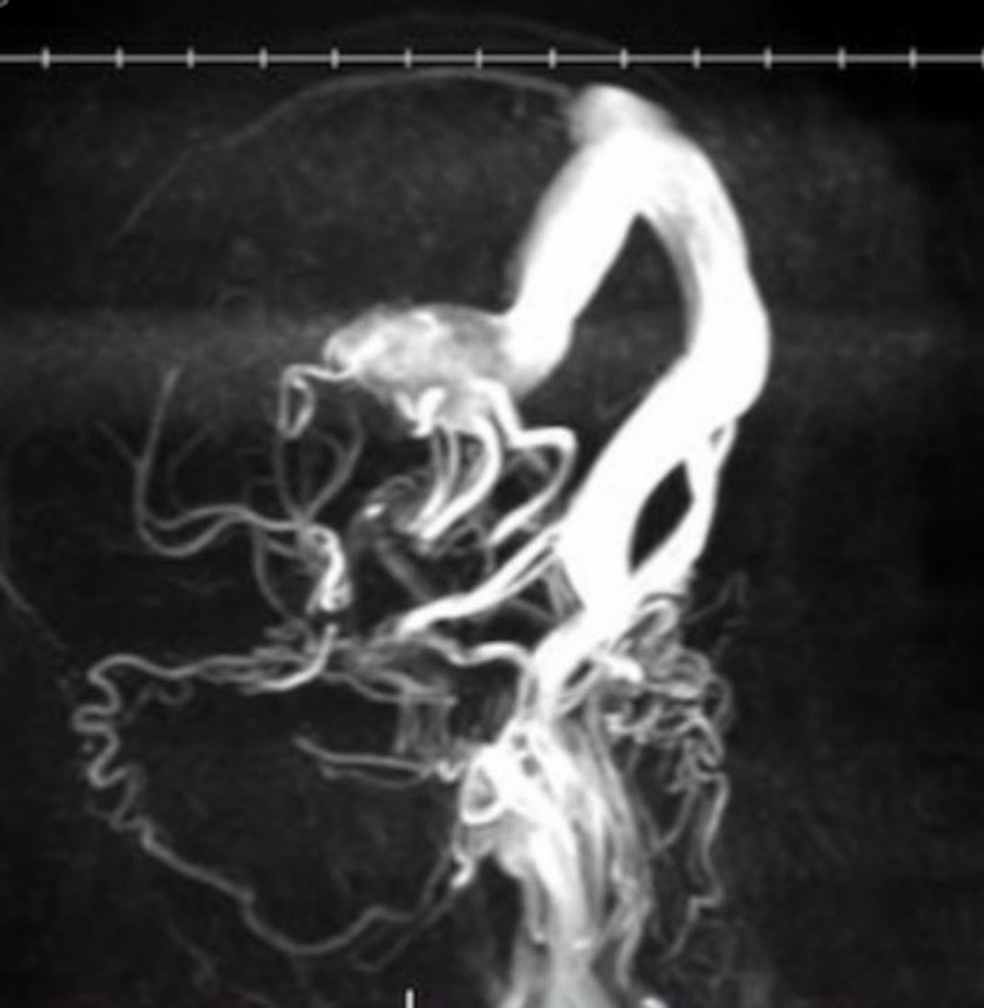
MRV of the brain showing the vein of Galen malformation Three-dimensional rotational projection views of MRV of the brain showing prominent bilateral posterior circulation and an enlarged midline vein draining into an enlarged straight sinus and superior sagittal sinus MRV, magnetic resonance venography

Head ultrasound showed normal ventricular sizes, symmetric cerebellar hemispheres, and no periventricular cysts or hemorrhages. However, it also showed increased color flow in a dilated vein of Galen, confirming the malformation. Magnetic resonance venography (Figure 1) showed bilateral prominent posterior cerebral and posterior communicating arteries (more enlarged on the right), and left anterior choroidal artery coursing into an enlarged midline vein which drained into an enlarged straight sinus and superior sagittal sinus. The enlarged midline vein measured up to 1.9 cm in greatest transverse diameter. Distended bilateral transverse and sigmoid sinuses and prominent tributary veins surrounding the enlarged midline vein were also noted.

The neurosurgery surgery was urgently consulted, and the patient was taken to the operating room. The overlying skin in the groin area around the right femoral artery was prepped and draped in a typical sterile fashion. The pulse was palpated and then the artery was punctured using a 4-French femoral sheath. Using cerebral angiography, a catheter was then carefully guided to the vein of Galen malformation. A coil was placed, followed by onyx glue for embolization. The catheter and femoral sheath were then removed, and the patient was sent back to the intensive care unit. Repeat imaging two days later showed an interval decrease in the size of the dilated midline vein. The patient gradually showed clinical improvement and was eventually extubated and successfully weaned to room air.

## Discussion

This infant presented with one day of oral intake intolerance and was found to be in heart failure due to the vein of Galen malformation. This case shows the importance of a proper workup for this seemingly simple chief complaint. Diagnoses to consider in a neonate with the acute onset of vomiting include pyloric stenosis, malrotation with volvulus, intussusception, duodenal web, foreign body ingestion (though less likely at this age), incarcerated hernia, gastroesophageal reflux disease, gastroenteritis (viral or bacterial), food protein-induced enteropathy, hydrocephalus, and meningitis. This clinical presentation coupled with the finding of heart failure in a neonate requires urgent management and a higher level of care. Heart failure can be the result of causes that are structural (such as valvular abnormalities) or functional (such as ventricular dysfunction due to volume overload). Neonates can present with poor feeding evidenced by cyanosis, sweating, and easy fatigability [5]. Thus, a proper history and physical examination are key to a successful diagnosis.

A head ultrasound is a relatively quick and cost-effective study that can be vital in the case of a neonate with the sudden onset of vomiting. In this case, it revealed our diagnosis of the vein of Galen malformation. The origin of this vein goes back embryologically to the formation of the anterior neuropore. As weeks in utero go by, the median prosencephalic vein of Markowski emerges. During weeks 6 to 11 in utero, the median prosencephalic vein is responsible for venous drainage from the developing fetal choroid plexus. As the typical embryological course continues, the vein of Markowski would eventually regress and the internal cerebral veins would take shape and continue drainage of the growing choroid plexus [3]. However, in the case of the congenital vein of Galen malformation, an arteriovenous shunt develops within the median prosencephalic vein instead. The shunt gives way to an increase in blood flow, making the median prosencephalic vein continuously dilated, and, thus, it persists and the regression that would have taken place is effectively prevented. The sequelae is abnormal anatomy of the dural venous sinuses due to increased vasculature and the postnatal vein of Galen malformation [6].

The clinical presentation of patients with the vein of Galen malformation varies based on age, but it most commonly manifests as high-output cardiac failure and/or hydrocephalus. At birth, the baby is separated from the placenta, a low-pressure system via which gas exchange occurs in the fetus. Upon this removal, there is an automatic increase in the baby's systemic vascular resistance and increased exposure to oxygen leads to vasodilation in pulmonary arteries and a subsequent decrease in the pulmonary vascular resistance. In patients with the vein of Galen malformation, the placenta and the arteriovenous shunt are both draining blood from the fetal choroid plexus in utero, but with removal of the placenta, there is decreased resistance and thus greater blood flow across the shunt [7]. This leads to increased venous return to the right atrium and thus increased preload, which can lead to pulmonary hypertension and congestive heart failure. The neonate can show signs of volume overload that is either responsive to diuretics or that can progress to cardiogenic shock.

In older infants and children, hydrocephalus and seizures are a more common clinical presentation. A dilated vein of Galen malformation can cause obstructive hydrocephalus by compression of the cerebral aqueduct, leading to cerebral venous congestion and abnormal cerebrospinal fluid (CSF) flow. These patients will have an enlarging head circumference with sunset eyes in severe cases, and they can have dilation of the scalp and facial veins and proptosis [6]. If the venous congestion is chronic due to late discovery, patients can go on to have developmental delay as they age. On the other hand, communicating hydrocephalus can occur due to the fact that a dilated vein of Galen causes poor formation of the dural sinuses and thus inadequate venous drainage. Increased blood flow to the arteriovenous shunt (with removal of the placenta) can result in decreased blood flow to the cerebral parenchyma and subsequent ischemic damage. These cause high pressure in the cerebral system leading to incomplete development of arachnoid granulation tissue and impaired CSF resorption, and, in severe cases, it leads to rapid loss of brain tissue, called the ‘melting brain syndrome’ [7].

Other complications of the vein of Galen malformation include myocardial ischemia and renal hypoperfusion. These can occur in patients with a particularly large arteriovenous malformation because the heart is working to pump a large volume to the lungs. Pulmonary hypertension results and decreases circulation to the rest of the body. This includes reduced coronary blood flow and subsequent increased myocardial oxygen requirements, as well as renal failure from lack of sufficient blood flow and a lactic acidosis [7].

Diagnosis of the vein of Galen malformation can occur prenatally on a fetal ultrasound, but this is not a common occurrence due to the low pressures in the venous system prior to birth and the fact that the placenta has not yet been removed. More often, it is diagnosed postnatally based on clinical presentation coupled with a subsequent head ultrasound. Other imaging modalities such as a computed tomography (CT) scan or magnetic resonance imaging (MRI) can be used to delineate the anatomy prior to treatment of this condition. A CT scan has its drawbacks because while it is effective in demonstrating the anatomy of the vessels of the shunt, one must use caution due to the risk of further renal damage with the requirement of contrast dye. MRI is thus more useful and less risky to the neonate for further analysis of this malformation and for adequate treatment planning [8].

In terms of the management of the vein of Galen malformation, cerebral angiography is both diagnostic and therapeutic; thus, it is the gold standard modality. It allows for precise detection of the anatomy surrounding the shunt and has the advantage of being able to detect the speed of blood flow within the vessels [9]. Embolization of the malformed vessel occurs via this method, effectively decreasing the flow of blood to the abnormal vasculature. When performed successfully, cerebral venous congestion begins to decrease, relieving pressure on the heart, and perfusion of the myocardium and kidneys show improvement toward the adequate level of flow. This procedure is not without complications, however. Some infants can develop limb ischemia when performed via the femoral artery route, which can result in leg asymmetry; others can develop emboli that can travel to the brain causing ischemia and subsequent neurological complications [10]. In recent years, however, the mortality rate has remarkably improved in those with high-output heart failure due to advances in endovascular embolization.

## Conclusions

The vein of Galen malformation should be considered in the differential diagnosis of any neonate who presents with the acute onset of vomiting. This diagnosis is rarely discovered prenatally, which put these patients at a greater risk of complications that can arise from delayed diagnosis. Underlined here is the importance of including a head ultrasound in the management of these patients as more common diagnoses are ruled out. This is a relatively easy study to obtain considering that the fontanels are readily accessible in the neonate. The clinical course of the patient with the vein of Galen malformation is largely dependent on timing of its discovery, and therefore care must be taken to rule this condition out as early as possible.

Importantly, dilation of the arteriovenous shunt is progressive and can rapidly lead to varying degrees of heart failure and other sequelae; therefore, these patients require a collaborative, multidisciplinary approach. Currently, the gold standard of treatment is endovascular embolization via cerebral angiogram. As the treatment modalities continue to advance, there will hopefully be a steeper decline in the mortality rate of this condition.

## References

[1] Bayot ML, Reddy V, Zabel MK (2021). Neuroanatomy, dural venous sinuses. StatPearls [Internet].

[2] San Millán Ruíz D, Gailloud P, Rüfenacht DA, Delavelle J, Henry F, Fasel JH (2002). The craniocervical venous system in relation to cerebral venous drainage. AJNR Am J Neuroradiol.

[3] Arko L, Lambrych M, Montaser A, Zurakowski D, Orbach DB (2020). Fetal and neonatal MRI predictors of aggressive early clinical course in vein of Galen malformation. AJNR Am J Neuroradiol.

[4] Recinos PF, Rahmathulla G, Pearl M, Recinos VR, Jallo GI, Gailloud P, Ahn ES (2012). Vein of Galen malformations: epidemiology, clinical presentations, management. Neurosurg Clin N Am.

[5] Das BB (2018). Current state of pediatric heart failure. Children (Basel).

[6] Bhattacharya JJ, Thammaroj J (2003). Vein of Galen malformations. J Neurol Neurosurg Psychiatry.

[7] Spazzapan P, Milosevic Z, Velnar T (2019). Vein of Galen aneurismal malformations - clinical characteristics, treatment and presentation: Three cases report. World J Clin Cases.

[8] Jones BV, Ball WS, Tomsick TA, Millard J, Crone KR (2002). Vein of Galen aneurysmal malformation: diagnosis and treatment of 13 children with extended clinical follow-up. AJNR Am J Neuroradiol.

[9] Gailloud P, O'Riordan DP, Burger I (2005). Diagnosis and management of vein of galen aneurysmal malformations. J Perinatol.

[10] Bhatia K, Mendes Pereira V, Krings T (2020). Factors contributing to major neurological complications from vein of Galen malformation embolization. JAMA Neurol.

